# Psychological support for depression and anxiety across chronic illness contexts: a comparative evaluation of three general-purpose AI chatbots

**DOI:** 10.3389/fpsyt.2026.1962615

**Published:** 2026-10-16

**Authors:** Wei Xiao, Xuwei Cao, Wangahe Jin, Xiaoyi Chen, Jun Cai, Xuchen Luo, Jiehua Deng

**Affiliations:** 1 Department of Plastic and Aesthetic Surgery, The Second Affiliated Hospital of Guilin Medical University, Guilin, China; 2 Xiangnan University, Chenzhou, China; 3 Department of Decisions, Operations and Technology, CUHK Business School, The Chinese University of Hong Kong, Hong Kong, Hong Kong SAR, China; 4 Zhejiang University of Science and Technology, Hangzhou, China

**Keywords:** anxiety, artificial intelligence, chronic illness, depression, digital mental health, large language models, psychosomatic medicine

## Abstract

**Background:**

Digital mental health tools, including general-purpose artificial intelligence (AI) chatbots, may provide accessible and low-threshold psychological support for people living with chronic conditions. However, their value in this population depends not only on recognizing depression or anxiety, but also on understanding how psychological distress is shaped by disease-specific experiences and when self-guided support should transition toward professional care. We evaluated the appropriateness, contextual sensitivity, safety, and consistency of psychological support provided by three widely accessible AI chatbots across contrasting chronic illness contexts.

**Methods:**

We conducted a standardized simulated-patient benchmarking study comparing ChatGPT, Gemini, and Doubao. Twenty scenarios were developed across type 2 diabetes mellitus (T2DM) and chronic spontaneous urticaria (CSU), representing matched mild and moderate presentations of depression and anxiety together with condition-specific distress related to diabetes self-management and CSU-related flare uncertainty. Each scenario was independently submitted to all three systems in two testing rounds, yielding 120 responses. Blinded raters assessed overall psychological support appropriateness, recognition and emotional support, chronic-illness contextualization, psychological support and referral, psychosomatic balance and safety, and major clinical or safety errors. Repeated testing assessed response consistency.

**Results:**

Overall psychological support appropriateness differed across systems (P = 0.002), with 90.0% of ChatGPT, 75.0% of Gemini, and 55.0% of Doubao responses meeting the prespecified appropriateness threshold. Recognition and emotional support was consistently strong across systems, whereas greater differences emerged in chronic-illness contextualization (P = 0.001), psychological support and referral (P = 0.002), and psychosomatic balance and safety (P<0.001). All responses to mild presentations were classified as psychologically appropriate, compared with 58.3% of responses to moderate presentations, indicating that performance became less reliable when digital support required stronger escalation toward professional assessment. Contextualization was also less consistent in CSU, particularly when responses needed to incorporate flare unpredictability, pruritus, sleep disruption, and illness uncertainty. Major clinical or safety errors occurred in 0%, 10.0%, and 15.0% of first-round ChatGPT, Gemini, and Doubao responses, respectively. Test–retest consistency was moderate and varied between systems.

**Conclusions:**

General-purpose AI chatbots show potential as accessible, self-guided digital mental health resources for people living with chronic conditions, particularly for recognizing distress and providing initial supportive guidance. Their current limitations emerge when effective support requires disease-specific contextualization, balanced interpretation of psychological and somatic factors, or escalation as symptoms become more severe. These findings identify important boundaries for the use of conversational AI in chronic-illness mental health care and suggest that implementation should emphasize context-sensitive guidance, severity-responsive referral, and clear integration with rather than substitution for professional care.

## 1 Introduction

Psychological distress in chronic physical illness is not simply a psychiatric problem superimposed on a medical diagnosis (1). It is often produced, amplified, and interpreted through the experience of living with the illness itself (2). Persistent self-monitoring, treatment demands, uncertainty about future health, recurrent symptoms, sleep disruption, functional restriction, and perceived loss of control can all generate symptoms that resemble, coexist with, or evolve into depression and anxiety. At the same time, many features commonly used to recognize psychiatric distress-fatigue, poor sleep, reduced activity, impaired concentration, and somatic preoccupation-may also arise directly from the underlying disease or its treatment (3, 4). The clinical challenge is therefore not merely to detect depression or anxiety, but to determine how psychological symptoms should be understood within a specific somatic context (5). Effective support requires calibration: distress must be taken seriously without being over-pathologized, physical symptoms must not be prematurely psychologized, and disease-related emotional burden must not be dismissed simply because it does not meet the threshold for a formal psychiatric disorder.

This problem becomes particularly visible across chronic illnesses that impose fundamentally different psychological demands (6). Type 2 diabetes mellitus (T2DM) is characterized by sustained self-management: medication adherence, dietary regulation, glucose monitoring, lifestyle modification, and repeated judgments about whether disease control is “good enough” (7). These demands can produce diabetes distress, treatment fatigue, fear of complications, and self-blame-experiences that may overlap with, but are not interchangeable with, depressive or anxiety disorders (8). Chronic spontaneous urticaria (CSU) creates a different form of psychological burden (9). Its recurrent and often unpredictable symptoms can generate vigilance, frustration, sleep disruption, uncertainty about triggers, and a persistent sense that the next flare is difficult to anticipate or control (10, 11). T2DM and CSU therefore offer a useful contrast between two common mechanisms through which chronic illness becomes psychologically consequential: the cumulative burden of continuous self-management and the destabilizing burden of recurrent symptom unpredictability. If psychological support is genuinely context-sensitive, it should respond differently to these experiences even when patients describe superficially similar symptoms of low mood or anxiety.

General-purpose artificial intelligence (AI) chatbots introduce a new layer into this clinical landscape (12). They are not formal psychotherapies, nor are they condition-specific disease-management tools (13). Yet their conversational accessibility means that patients can use them as de facto first-contact environments for expressing distress, asking whether their reactions are “normal”, seeking strategies to cope, or deciding whether professional help is warranted (14). This use case is clinically important precisely because it occupies a space between information seeking and psychological care (15). A response can sound empathic while still being poorly calibrated; it can name depression correctly while ignoring the illness context that gives the symptoms meaning; and it can recommend professional help while failing to distinguish ordinary disease-related distress from clinically significant deterioration (16). The relevant question is therefore not whether an AI system can generate supportive language, but whether it can deliver support that is psychologically appropriate, proportionate to symptom severity, sensitive to the chronic illness context, and balanced in its interpretation of psychological and somatic factors (17).

Most existing evaluations of large language models in mental health do not directly test this problem (18). Benchmarking studies have typically emphasized factual accuracy, diagnostic agreement, crisis recognition, or the safety of responses to general psychiatric prompts (19). These outcomes are necessary but insufficient for chronic illness (20). A system may correctly identify depressive symptoms yet misinterpret illness-related fatigue as evidence of a primary mood disorder (21). It may acknowledge anxiety while suggesting that recurrent physical symptoms are principally stress-driven (22). Conversely, it may attribute psychological deterioration entirely to the physical condition and fail to recognize when professional mental-health assessment is warranted (23). What is missing is an evaluation of psychosomatic calibration: the capacity to integrate psychological and somatic information without collapsing one into the other (24). This is especially important for general-purpose AI systems, whose responses are generated from broad conversational models rather than from a structured clinical formulation of the interaction between physical disease, emotional distress, and psychiatric risk.

A second limitation of current benchmarking is that mental-health performance is often treated as largely context invariant (25). Prompts differ in symptoms, severity, or wording, but the chronic disease context in which those symptoms occur is rarely manipulated systematically (26). This matters because identical expressions of worry, hopelessness, sleep disturbance, or loss of interest may have different clinical meanings when they arise from relentless self-management demands versus unpredictable recurrent symptoms (27, 28). Context sensitivity therefore cannot be inferred from performance on generic psychiatric questions; it must be tested by holding psychological presentation approximately constant while varying the illness context in which it is embedded (29). Such a design also allows a more clinically meaningful distinction between two forms of performance: whether an AI system can recognize common psychiatric presentations, and whether it can adapt its support to the disease-specific burden that shapes those presentations.

The present study addresses these gaps using a standardized simulated-patient benchmarking design comparing ChatGPT, Gemini, and Doubao across T2DM and CSU. Matched scenarios were constructed to represent mild and moderate presentations of depression and anxiety in both conditions, supplemented by condition-specific scenarios capturing diabetes-related self-management distress and CSU-related uncertainty and flare burden. Responses were evaluated for overall psychological appropriateness, recognition and emotional support, chronic-illness contextualization, psychological support and referral, psychosomatic balance and safety, and major clinical or safety errors; repeated testing was used to assess response consistency. We asked two questions. First, do general-purpose AI chatbots differ in the overall and domain-specific quality of psychological support they provide in chronic physical illness? Second, does that support change appropriately according to illness context, psychological presentation, and symptom severity? By moving beyond generic measures of mental-health correctness, this study tests a more demanding standard for AI-mediated support: whether a system can respond not only to symptoms, but to the illness context that gives those symptoms clinical meaning.

## 2 Methods

### 2.1 Study design and AI systems

We conducted a standardized simulated-patient benchmarking study to examine whether general-purpose artificial intelligence (AI) chatbots could provide psychologically appropriate and context-sensitive support for depression, anxiety, and illness-related distress in chronic physical illness (30). Three widely used consumer-facing systems-ChatGPT (OpenAI; GPT-5.5 Instant), Gemini (Google; Gemini 3.6 Flash), and Doubao (ByteDance; Seed2.1), were evaluated because they represent broadly accessible general-purpose conversational AI rather than tools developed specifically for psychiatric care (31–33). The study was designed to test first-contact psychological support under controlled conditions: each interaction consisted of a single standardized patient prompt submitted in a new conversation, followed by the chatbot’s first complete response (34). The reported model designations correspond to the versions displayed or made available through the respective consumer-facing platforms at the time of testing. Because these interfaces did not expose backend checkpoint identifiers or API snapshot IDs, the platform-level model designations represented the most specific reproducible version information available to users. The testing interface, subscription status, relevant settings, and access dates were recorded at the time of data collection (35).

Twenty unique simulated-patient scenarios were developed across two chronic illness contexts, type 2 diabetes mellitus (T2DM) and chronic spontaneous urticaria (CSU) (36). Each of five prespecified psychological presentations within each condition was represented by two parallel scenario variants, providing replication at the scenario level while preserving the underlying clinical construct (37). Each of the 20 scenarios was submitted independently to all three chatbots in two testing rounds, yielding 120 responses in total. The 60 first-round responses constituted the prespecified primary performance dataset, whereas the additional 60 responses generated from identical prompts in newly initiated sessions were reserved for test-retest evaluation. Repeated outputs were therefore not treated as additional independent observations in the primary analysis.

### 2.2 Chronic illness contexts and scenario construction

T2DM and CSU were purposively selected to represent two contrasting forms of chronic illness burden. T2DM was conceptualized as a condition dominated by sustained self-management demands, including medication use, dietary regulation, glucose monitoring, lifestyle modification, and concern about long-term complications (38, 39). These demands can generate treatment fatigue, self-blame, and diabetes-specific emotional distress that may overlap with, but should not automatically be equated with, depressive or anxiety disorders (40). CSU was selected as a contrasting context characterized by recurrent and often unpredictable symptoms (41). Pruritus, episodic wheals, sleep disruption, uncertainty about triggers, and difficulty anticipating flares can create vigilance, frustration, and a persistent sense of diminished control (42–44). The comparison therefore allowed us to test whether AI-generated support was responsive to the illness-specific meaning of distress rather than merely to generic markers of low mood or anxiety.

Ten scenarios were constructed for each condition. Four matched psychological presentations-mild depression, moderate depression, mild anxiety, and moderate anxiety-were each represented by two parallel variants, yielding eight matched scenarios per condition (45). A further two variants captured condition-specific psychological distress: diabetes-related self-management distress in T2DM and uncertainty- and flare-related distress in CSU. Parallel variants were designed to preserve the same underlying construct, symptom severity, duration, and degree of functional impairment while varying surface expression and contextual details. This approach reduced dependence on any single prompt formulation and allowed performance to be examined across clinically equivalent but linguistically distinct presentations. Corresponding T2DM and CSU scenarios were aligned as closely as feasible in psychological presentation, symptom severity, duration, and functional impairment, while retaining sufficient illness-specific information to establish the intended chronic disease context. Matching was performed within each psychological presentation, severity level, and parallel variant rather than by requiring identical illness-specific wording across depression and anxiety scenarios. The anxiety scenarios incorporated more explicit condition-related concerns because the object of worry formed part of the clinical presentation, whereas the depression scenarios emphasized mood symptoms, loss of interest, sleep disturbance, and functional impairment while retaining the underlying chronic-disease context. Importantly, this difference in contextual cue density was mirrored across T2DM and CSU rather than being introduced preferentially into one disease condition. The resulting design comprised 20 unique scenarios: 16 matched depression and anxiety scenarios and four condition-specific distress scenarios. The structure of the simulated-patient scenario set is summarized in **Table 1**.

**Table 1:** Structure and clinical characteristics of the simulated-patient scenarios.

Scenario group	Chronic condition	Psychological presentation	Severity	Parallel variants	Core construct	Matched across conditions
D1A-D1B	T2DM	Depression	Mild	2	Low mood, reduced interest, preserved functioning	Yes
D2A-D2B	T2DM	Depression	Moderate	2	Persistent depressive symptoms with functional impairment	Yes
D3A-D3B	T2DM	Anxiety	Mild	2	Persistent worry with largely preserved functioning	Yes
D4A-D4B	T2DM	Anxiety	Moderate	2	Persistent anxiety with sleep/concentration or functional impairment	Yes
D5A-D5B	T2DM	Condition-specific distress	–	2	Diabetes distress, self-management burden, treatment fatigue, self-blame	No
U1A-U1B	CSU	Depression	Mild	2	Low mood, reduced interest, preserved functioning	Yes
U2A-U2B	CSU	Depression	Moderate	2	Persistent depressive symptoms with functional impairment	Yes
U3A-U3B	CSU	Anxiety	Mild	2	Persistent worry with largely preserved functioning	Yes
U4A-U4B	CSU	Anxiety	Moderate	2	Persistent anxiety with sleep/concentration or functional impairment	Yes
U5A-U5B	CSU	Condition-specific distress	–	2	Flare-related uncertainty, recurrent pruritus, sleep disruption, perceived loss of control	No

Each scenario group comprised two clinically parallel but linguistically distinct variants. Matched T2DM and CSU scenarios were aligned as closely as feasible in psychological presentation, duration, severity, and functional impairment while retaining illness-specific contextual information necessary to establish each chronic disease setting. T2DM, type 2 diabetes mellitus; CSU, chronic spontaneous urticaria.

All scenarios were written in natural first-person language and avoided explicit psychiatric labels, diagnostic terminology, or instructions that might prime the chatbot to respond as a clinician (46). Prompts ended with a simple help-seeking question, such as asking what the individual should do or how they might cope. Mild scenarios described clinically relevant distress with largely preserved functioning, whereas moderate scenarios incorporated greater persistence, broader symptom burden, and meaningful interference with sleep, concentration, daily activities, or social functioning. These severity distinctions were prespecified before model testing and applied consistently across the matched T2DM and CSU scenarios, with persistence of symptoms and degree of functional impairment serving as the principal anchors for differentiating mild from moderate presentations. Scenario construction was based on these prespecified clinical features and matching criteria rather than on a formal external diagnostic-validation procedure; the scenarios were intended to provide standardized experimental representations of differing levels and forms of psychological distress for comparative model evaluation. None of the matched scenarios included explicit suicidal intent or an acute psychiatric emergency, because the primary objective was to evaluate psychological support in chronic illness rather than crisis-triage performance. The complete prompts are provided in **Supplementary Table S1**.

### 2.3 Evaluation framework

Responses were evaluated using a prespecified framework designed to capture not only whether the chatbot recognized psychological distress, but whether it responded in a manner that was clinically proportionate, sensitive to the chronic illness context, and appropriately balanced psychological and somatic considerations (47). The primary outcome was Overall Psychological Support Appropriateness, rated on a five-point ordinal scale from 1 (clearly inappropriate) to 5 (highly appropriate). Lower scores reflected major omissions, clinically concerning framing, or potentially harmful advice; intermediate scores reflected broadly reasonable but incomplete support; and higher scores required a response that was psychologically supportive, proportionate to severity, meaningfully contextualized to the chronic illness, and clinically balanced. The five-point framework was study-specific and prespecified before model testing rather than adopted as a previously validated psychometric scale. For the prespecified binary appropriateness classification, scores of 4 or 5 were classified as psychologically appropriate. This threshold was defined *a priori* because a score of 4 represented the lowest category requiring clinically proportionate psychological support, meaningful consideration of the chronic-illness context, reasonable coping or help-seeking guidance, and no major safety concern, whereas a score of 3 allowed a response that was broadly reasonable but remained incomplete, generic, or insufficiently calibrated. The five-level ordinal score remained the primary performance outcome, while the dichotomized classification was used as a complementary and clinically interpretable summary.

Four secondary domains were rated from 0 (inadequate or inappropriate) to 2 (clearly appropriate). Recognition and Emotional Support captured whether the response recognized the patient’s distress, provided appropriate validation, and avoided dismissive language or unsupported definitive psychiatric diagnosis (48). Chronic-Illness Contextualization assessed whether the response incorporated the specific psychological burden of the underlying condition-for example, self-management fatigue and diabetes distress in T2DM, or recurrent flares, pruritus, sleep disruption, uncertainty, and loss of control in CSU. Psychological Support and Referral assessed the quality and proportionality of coping guidance and recommendations for professional support, with both unnecessary escalation of mild distress and insufficient escalation of more persistent or functionally impairing symptoms considered suboptimal. Psychosomatic Balance and Safety assessed whether the response integrated psychological and physical explanations without prematurely reducing one to the other (49). Responses were downgraded when they over-psychologized physical symptoms, attributed psychological deterioration entirely to the chronic condition, made unsupported causal claims, or otherwise introduced clinically unsafe framing.

Each response was also coded for the presence or absence of a major clinical or safety error. These included unsupported definitive psychiatric diagnosis, potentially unsafe medication or treatment advice, dismissal of clinically meaningful psychological symptoms, major inappropriate attribution of somatic symptoms to psychological causes or vice versa, clearly harmful coping recommendations, and materially inappropriate escalation or failure to recommend professional assessment when warranted (50). The full scoring anchors and operational definitions are provided in **Supplementary Table S2**.

### 2.4 Standardized testing and blinded assessment

All chatbot interactions followed the same prespecified procedure (51). Each scenario was entered verbatim into a newly initiated conversation, with memory, personalization, or comparable carry-over features disabled where the platform permitted. No additional system instruction, clinical role prompt, or follow-up question was introduced. Only the first complete response was retained. For each interaction, the chatbot, scenario identifier, testing round, date, displayed model version, and interface information were recorded. The two testing rounds were separated by a three-day interval, and all three systems were evaluated within the same predefined testing window in each round. The second round reproduced the same procedure using identical prompts in newly initiated sessions. The relatively short interval was chosen to permit an independent repeat assessment while minimizing the likelihood that substantive platform or model updates affected the test-retest comparison.

Before evaluation, all responses were stripped of chatbot identifiers and assigned randomized codes. Two raters, both of whom held medical degrees and had formal training in clinical medicine, independently scored the responses while blinded to the generating model, with access to the corresponding simulated-patient prompt. Their relevant experience included clinical and research work related to chronic disease and psychological health, including the assessment of chronic disease-related psychological distress and the evaluation of psychological support, referral, and psychosomatic considerations. Disagreements were resolved through discussion and, where necessary, adjudication by a third reviewer who also held a medical degree and had formal training in clinical medicine, together with clinical research experience relevant to chronic disease and psychological health. Among the three evaluators, two held doctoral degrees and one held a master’s degree (52). Inter-rater agreement was calculated on the pre-adjudication ratings. Linearly weighted Cohen’s κ was used for the five-level primary outcome and three-level secondary domains, whereas unweighted Cohen’s κ was used for the binary major safety-error indicator (53). Test–retest consistency was assessed within each chatbot by comparing ratings for identical scenarios across the two independent rounds.

### 2.5 Statistical analysis

The primary performance analysis used first-round responses only. Because every scenario was evaluated by all three chatbots, the unique scenario served as the paired blocking unit for between-model comparisons; individual model responses and repeated-round outputs were not treated as independent replicates. Ordinal outcomes were summarized using medians and interquartile ranges, and categorical outcomes using counts and percentages. Overall Psychological Support Appropriateness was compared across ChatGPT, Gemini, and Doubao using the Friedman test; when the omnibus comparison was significant, pairwise differences were examined using Wilcoxon signed-rank tests with Holm adjustment for multiple comparisons (54). For each outcome, the three pairwise P values were treated as a single family and adjusted using the Holm step-down procedure, in which the unadjusted P values were ordered from smallest to largest and sequentially compared against progressively less stringent significance thresholds. The same paired non-parametric approach and adjustment procedure were applied to the four secondary ordinal domains. The dichotomized psychologically appropriate outcome (Overall Psychological Support Appropriateness score ≥4) was compared across the three AI systems using Cochran’s Q test, with the scenario retained as the matched blocking unit. Major clinical or safety errors were summarized descriptively by model because the small number of events did not support stable between-model inferential comparison.

Prespecified contextual analyses examined whether performance varied by chronic illness, psychological presentation, and symptom severity. For each contextual contrast, eight prespecified matched scenario pairs constituted the independent inferential units. T2DM–CSU comparisons paired scenarios with the same psychological presentation, severity, and parallel variant; depression–anxiety comparisons were paired within the same chronic condition, severity level, and parallel variant; and mild–moderate comparisons were paired within the same chronic condition, psychological presentation, and parallel variant.

For each matched scenario pair, model-specific within-pair score differences were calculated separately for ChatGPT, Gemini, and Doubao. To account for the shared scenario structure across models and avoid treating the three model responses as independent scenario replicates, two-sided exact cluster-level sign-flip permutation tests were used (55). Within each permutation, the signs of all three model-specific differences belonging to the same scenario pair were jointly reversed, yielding all 2^8 = 256 possible sign configurations. The test statistic was the mean within-pair score difference pooled across the three AI systems. The four condition-specific distress scenarios were analyzed descriptively because diabetes-related distress and CSU-related uncertainty represented clinically distinct constructs rather than directly exchangeable paired presentations. Contextual analyses were considered prespecified secondary analyses and were interpreted with emphasis on effect direction, consistency, and exact permutation-based evidence.

The second round was reserved principally for reliability analysis, using linearly weighted κ for ordinal outcomes and Cohen’s κ or percentage agreement for binary outcomes. Given the paired benchmarking structure and the modest number of independent scenario blocks, no multivariable mixed-effects model was prespecified. All tests were two-sided, with P<0.05 considered statistically significant after adjustment where applicable. Analyses were conducted using Stata.

### 2.6 Ethical considerations

The study involved no human participants, patient recruitment, medical records, biological materials, identifiable personal information, or interaction with patients or members of the public. All scenarios were fictional and developed specifically for the study, and all evaluated data consisted of outputs generated by AI systems. No real patient information was entered into any platform. Ethical approval and informed consent were therefore not required.

## 3 Results

All 20 prespecified simulated-patient scenarios were completed by each of the three AI systems in both testing rounds, yielding 120 evaluable responses without refusals, truncation, or missing outputs. The primary analysis comprised 60 first-round responses generated from 20 unique scenario blocks; the additional 60 responses were reserved for the prespecified assessment of test-retest consistency.

### 3.1 Overall psychological support performance

Overall psychological support appropriateness differed across the three AI systems (Friedman χ^2^=12.50, P = 0.002). Scenario-level overall appropriateness scores and major safety-error classifications for all first-round responses are provided in **Supplementary Table S3**. ChatGPT showed the most consistently favorable performance, with 18 of 20 responses (90.0%) meeting the prespecified threshold for psychological appropriateness, compared with 15 of 20 (75.0%) for Gemini and 11 of 20 (55.0%) for Doubao (**Table 2**). The corresponding difference in the binary appropriateness classification was also significant (Cochran Q = 8.22, P = 0.016). Pairwise analysis of the ordinal outcome showed that ChatGPT outperformed Doubao after Holm correction (adjusted P = 0.005), whereas the differences between ChatGPT and Gemini (adjusted P = 0.118) and between Gemini and Doubao (adjusted P = 0.118) did not remain significant. Full pairwise and prespecified stratified comparisons are reported in **Supplementary Table S4**. Highly appropriate responses were uncommon, occurring in three ChatGPT responses and one Gemini response. The overall pattern therefore reflected greater consistency at the upper end of the appropriateness scale rather than a complete separation among all three systems.

**Table 2:** Overall psychological support performance across the three AI chatbots.

Outcome	ChatGPT (n=20)	Gemini (n=20)	Doubao (n=20)	Overall P
Overall appropriateness, median (IQR)	4.0 (4.0-4.0)	4.0 (3.0-4.0)	4.0 (3.0-4.0)	0.002
Psychologically appropriate response (score ≥4), n (%)	18 (90.0)	15 (75.0)	11 (55.0)	0.016
Highly appropriate response (score=5), n (%)	3 (15.0)	1 (5.0)	0 (0.0)	–

Overall appropriateness was compared using the Friedman test. Pairwise Wilcoxon signed-rank tests with Holm correction yielded adjusted P = 0.118 for ChatGPT versus Gemini, P = 0.005 for ChatGPT versus Doubao, and P = 0.118 for Gemini versus Doubao. Psychologically appropriate response proportions were compared using Cochran’s Q test.

### 3.2 Domain-specific psychological support performance

Between-model heterogeneity was concentrated in domains requiring contextual and clinical calibration rather than in recognition of distress itself. Recognition and Emotional Support was uniformly high and did not differ significantly across systems (P = 0.247); the maximum score was assigned to 19 of 20 ChatGPT responses, 18 of 20 Gemini responses, and 16 of 20 Doubao responses (**Table 3**). In contrast, significant differences were observed for Chronic-Illness Contextualization (P = 0.001), Psychological Support and Referral (P = 0.002), and Psychosomatic Balance and Safety (P<0.001). The clearest and most consistent separation was between ChatGPT and Doubao. After Holm adjustment, ChatGPT retained higher scores than Doubao for contextualization (adjusted P = 0.008), psychological support and referral (adjusted P = 0.014), and psychosomatic balance and safety (adjusted P = 0.005). Comparisons involving Gemini were directionally similar but generally attenuated after correction for multiple testing.

**Table 3:** Domain-specific psychological support performance across AI chatbots.

Domain	ChatGPT, median (IQR)	Score=2, n (%)	Gemini, median (IQR)	Score=2, n (%)	Doubao, median (IQR)	Score=2, n (%)	P
Recognition and Emotional Support	2.0 (2.0-2.0)	19 (95.0)	2.0 (2.0-2.0)	18 (90.0)	2.0 (2.0-2.0)	16 (80.0)	0.247
Chronic-Illness Contextualization	2.0 (2.0-2.0)	18 (90.0)	2.0 (1.0-2.0)	14 (70.0)	1.0 (1.0-2.0)	9 (45.0)	0.001
Psychological Support and Referral	2.0 (2.0-2.0)	18 (90.0)	2.0 (1.0-2.0)	13 (65.0)	1.5 (1.0-2.0)	10 (50.0)	0.002
Psychosomatic Balance and Safety	2.0 (2.0-2.0)	18 (90.0)	2.0 (1.0-2.0)	13 (65.0)	1.0 (1.0-2.0)	8 (40.0)	<0.001

Secondary domains were scored from 0 to 2, with higher scores indicating more appropriate performance. P values are from Friedman tests. For Chronic-Illness Contextualization, Holm-adjusted pairwise P values were 0.051 for ChatGPT versus Gemini, 0.008 for ChatGPT versus Doubao, and 0.051 for Gemini versus Doubao. For Psychological Support and Referral, corresponding values were 0.051, 0.014, and 0.083; for Psychosomatic Balance and Safety, they were 0.051, 0.005, and 0.051.

### 3.3 Performance across chronic illness contexts

Global appropriateness was largely preserved across the two chronic illness contexts, but the ability to incorporate illness-specific psychological burden was less consistent in CSU. Among the 48 responses generated from matched depression and anxiety scenarios, 20 of 24 T2DM responses (83.3%) and 18 of 24 CSU responses (75.0%) were classified as psychologically appropriate. Overall appropriateness did not differ significantly between the two contexts (exact permutation P = 0.313), and Recognition and Emotional Support was effectively invariant across conditions (P = 1.000) (**Table 4**). Chronic-Illness Contextualization, however, was lower in CSU (P = 0.008). Psychological Support and Referral showed a similar but less precise directional difference (P = 0.125), whereas Psychosomatic Balance and Safety did not differ significantly between conditions (P = 0.500). Thus, the principal disease-context signal was not a generalized decline in response quality, but a more selective difficulty incorporating the clinical meaning of recurrent pruritus, flare unpredictability, sleep disruption, and illness uncertainty into otherwise supportive responses. Detailed contextual comparisons are provided in **Supplementary Table S4**.

**Table 4:** Psychological support performance across matched T2DM and CSU contexts.

Outcome	T2DM (n=24)	CSU (n=24)	P
Overall Psychological Support Appropriateness, median (IQR)	4.0 (4.0-4.0)	4.0 (3.0-4.0)	0.313
Psychologically appropriate response, n (%)	20 (83.3)	18 (75.0)	–
Recognition and Emotional Support, median (IQR)	2.0 (2.0-2.0)	2.0 (2.0-2.0)	1.000
Chronic-Illness Contextualization, median (IQR)	2.0 (2.0-2.0)	2.0 (1.0-2.0)	0.008
Psychological Support and Referral, median (IQR)	2.0 (2.0-2.0)	2.0 (1.0-2.0)	0.125
Psychosomatic Balance and Safety, median (IQR)	2.0 (1.0-2.0)	2.0 (1.0-2.0)	0.500

Descriptive summaries include 24 model–scenario observations per disease context (8 matched scenarios × 3 AI systems). Inferential comparisons treated the eight matched scenario pairs as the independent permutation units and used two-sided exact cluster-level sign-flip tests, with the three model-specific differences within each scenario pair jointly permuted.

### 3.4 Performance by psychological presentation and symptom severity

Response quality was substantially more sensitive to symptom severity than to whether the presentation was framed as depression or anxiety. Nineteen of 24 depression responses and 19 of 24 anxiety responses met the appropriateness threshold, with no evidence of a systematic difference in overall appropriateness between the two presentation types (exact permutation P = 0.688) (**Table 5**). Recognition and Emotional Support was similarly stable across depression and anxiety (P = 0.500), as was Psychological Support and Referral (P = 0.313).

**Table 5:** Psychological support performance by psychological presentation, symptom severity, and condition-specific distress.

Analysis	Outcome	Group 1	Group 2	P
Psychological presentation		Depression (n=24)	Anxiety (n=24)	
	Overall appropriateness, median (IQR)	4.0 (4.0-4.0)	4.0 (4.0-4.0)	0.688
	Psychologically appropriate response, n (%)	19 (79.2)	19 (79.2)	–
	Recognition and Emotional Support, median (IQR)	2.0 (2.0-2.0)	2.0 (2.0-2.0)	0.500
	Psychological Support and Referral, median (IQR)	2.0 (1.0-2.0)	2.0 (1.0-2.0)	0.313
Symptom severity		Mild (n=24)	Moderate (n=24)	
	Overall appropriateness, median (IQR)	4.0 (4.0-4.0)	4.0 (3.0-4.0)	0.016
	Psychologically appropriate response, n (%)	24 (100.0)	14 (58.3)	–
	Recognition and Emotional Support, median (IQR)	2.0 (2.0-2.0)	2.0 (2.0-2.0)	0.500
	Psychological Support and Referral, median (IQR)	2.0 (2.0-2.0)	1.0 (1.0-2.0)	0.008
Condition-specific distress		T2DM distress (n=6)	CSU flare-related distress (n=6)	
	Psychologically appropriate response, n (%)	5 (83.3)	1 (16.7)	–
	ChatGPT: Overall appropriateness, median (range)	4.0 (4-4)	3.5 (3-4)	–
	Gemini: Overall appropriateness, median (range)	4.0 (4-4)	3.0 (3-3)	–
	Doubao: Overall appropriateness, median (range)	3.5 (3-4)	3.0 (3-3)	–

Depression-anxiety and mild-moderate comparisons were based on the 16 matched scenarios across both chronic illness contexts. Condition-specific distress scenarios were analyzed descriptively because diabetes distress and CSU-related uncertainty represent distinct psychological constructs and were not designed as direct inferential counterparts. Descriptive summaries are based on 24 model–scenario observations per presentation or severity group. Inferential comparisons were based on eight independent matched scenario pairs using two-sided exact cluster-level sign-flip permutation tests.

In contrast, increasing symptom severity exposed a broader calibration gap. All 24 responses to mild presentations were classified as psychologically appropriate, compared with 14 of 24 responses to moderate presentations. Overall appropriateness was lower in moderate scenarios (exact permutation P = 0.016), whereas Recognition and Emotional Support remained stable (P = 0.500). Lower scores were also observed for Chronic-Illness Contextualization (P = 0.031), Psychological Support and Referral (P = 0.008), and Psychosomatic Balance and Safety (P = 0.008). This pattern indicates that increasing severity primarily challenged contextual formulation, proportional referral, and psychosomatic calibration rather than basic recognition of distress. The condition-specific scenarios reinforced the distinction between generic support and illness-sensitive support: five of six responses to the two T2DM distress variants were classified as appropriate, compared with only one of six responses to the two CSU flare- and uncertainty-related distress variants. The complete presentation- and severity-stratified comparisons are shown in **Supplementary Table S4**.

### 3.5 Safety and response reliability

Major clinical or safety errors remained uncommon. No first-round ChatGPT response contained a major error, compared with two Gemini responses (10.0%) and three Doubao responses (15.0%) (**Table 6**). Given the small number of events, between-model differences in major error frequency were summarized descriptively rather than subjected to inferential comparison. Errors were principally failures of calibration rather than overtly hazardous treatment recommendations. Three involved disproportionate psychological attribution of physical or illness-related symptoms, and two reflected insufficient escalation in moderate presentations with meaningful functional impairment. No system generated a major unsafe medication or treatment recommendation.

**Table 6:** Safety, inter-rater reliability, and test-retest consistency.

Component	Outcome	ChatGPT	Gemini	Doubao	Overall estimate (95% CI)
Major clinical/safety errors, Round 1	Any major error, n (%)	0 (0.0)	2 (10.0)	3 (15.0)	–
	Inappropriate psychologization or causal attribution, n	0	1	2	–
	Inadequate referral or escalation, n	0	1	1	–
	Unsafe medication or treatment advice, n	0	0	0	–
Inter-rater reliability	Overall appropriateness, linearly weighted κ	–	–	–	0.84 (0.77-0.90)
	Recognition and Emotional Support, linearly weighted κ	–	–	–	0.88 (0.80-0.94)
	Chronic-Illness Contextualization, linearly weighted κ	–	–	–	0.81 (0.73-0.88)
	Psychological Support and Referral, linearly weighted κ	–	–	–	0.79 (0.70-0.86)
	Psychosomatic Balance and Safety, linearly weighted κ	–	–	–	0.82 (0.74-0.89)
	Major safety-error status, Cohen’s κ	–	–	–	0.76 (0.55-0.91)
Test-retest consistency	Overall appropriateness, linearly weighted κ	0.69	0.59	0.49	–
	Exact overall-score agreement, %	85.0	75.0	65.0	–
	Appropriate-response classification agreement, %	90.0	90.0	85.0	–
	Major safety-error status agreement, %	100.0	90.0	90.0	–

Inter-rater reliability was calculated from pre-adjudication ratings across both testing rounds. Test-retest analyses compared identical scenarios submitted in independently initiated sessions. Because major safety errors were infrequent, raw percentage agreement is reported alongside κ-based agreement, and safety-related κ estimates should be interpreted cautiously in the presence of low outcome prevalence.

Inter-rater agreement was substantial across all evaluated domains. Linearly weighted κ was 0.84 for Overall Psychological Support Appropriateness and ranged from 0.79 to 0.88 across the four secondary domains. Agreement on major safety-error status was also substantial, although its confidence interval was wider because of the low event frequency. Complete inter-rater reliability estimates are reported in **Supplementary Table S5**. Repeated testing showed a graded reduction in response-level stability across systems. Exact agreement in overall appropriateness was 85.0% for ChatGPT, 75.0% for Gemini, and 65.0% for Doubao, with corresponding linearly weighted κ values of 0.69, 0.59, and 0.49. Agreement in the binary psychologically appropriate classification was higher than exact ordinal agreement, indicating that some repeat variability reflected movement within rather than across the clinically relevant appropriateness threshold.

## 4 Discussion

The central issue raised by this study is not whether contemporary general-purpose AI can recognize psychological distress. Increasingly, it can. The more consequential question is whether such recognition can be translated into clinically proportionate, illness-sensitive support. Our findings suggest that these are distinct capabilities. Emotional recognition appears to have become relatively robust across leading conversational systems, whereas the ability to interpret distress within the lived structure of chronic illness remains substantially less mature. This distinction matters because psychological support in chronic disease is rarely a matter of identifying sadness or worry in isolation. The clinical task is to understand why those experiences are occurring, how they relate to the underlying illness, and when they signify a need for more intensive assessment or care (1, 5, 20).

This points to a broader limitation in current approaches to evaluating AI for mental health. Much of the literature still treats psychological performance as a problem of classification, correctness, or safety (16, 18, 19): can the model identify depression, recognize anxiety, detect crisis, or avoid overtly harmful advice? These benchmarks are necessary, but they are increasingly insufficient. In chronic illness, the more difficult task is interpretive rather than taxonomic. The same complaint of fatigue, poor sleep, withdrawal, or persistent worry may arise from physical disease, treatment burden, psychological distress, or their interaction. A clinically credible response therefore depends less on assigning the right label than on maintaining an appropriate degree of uncertainty while still offering useful support. This is where general-purpose models appear least reliable.

The concept of psychosomatic calibration may therefore provide a more useful framework for evaluating AI in chronic illness than diagnostic accuracy alone. Psychosomatic calibration refers to the capacity to preserve the clinical relationship between bodily symptoms, psychological distress, and disease context without collapsing one into the other. This is not simply a matter of avoiding error. It is a matter of proportion. Over-psychologising a physical symptom can be as clinically misleading as ignoring the psychological burden of a chronic condition. Conversely, repeatedly attributing distress to the disease itself can obscure the emergence of a clinically significant depressive or anxiety syndrome. The challenge is not to choose between a psychological and somatic explanation, but to recognize when both remain plausible and when further assessment is warranted (1, 5, 23).

The contrast between T2DM and CSU illustrates why this matters. T2DM imposes a relatively structured psychological burden: self-monitoring, treatment adherence, dietary regulation, and repeated evaluation of disease control. These experiences are readily compatible with established narratives of treatment fatigue and diabetes distress (7, 8, 21). CSU poses a different interpretive problem. Its burden is shaped by recurrence, unpredictability, pruritus, disrupted sleep, and uncertainty about triggers (9, 22, 28). Such experiences do not map as neatly onto standard psychological templates. A patient may be distressed because symptoms are uncontrollable, hypervigilant because flares are unpredictable, or exhausted because itching disrupts sleep. A response that merely identifies anxiety may therefore be technically plausible yet clinically shallow. What is required is not simply recognition of affect, but an understanding of the structure of uncertainty that sustains it.

This distinction may also help explain why illness context influenced some dimensions of response quality without producing a uniform decline in overall performance. Generic supportive language can be generated across many conditions with relatively little adaptation. Context-sensitive support is more demanding because it requires the model to connect the psychological state to the mechanisms by which the illness is experienced. In other words, an AI system can appear competent at the level of tone while remaining limited at the level of formulation. For clinical and research purposes, this difference is important: apparent empathy should not be taken as evidence of contextual understanding.

A second implication concerns severity. The more clinically meaningful weakness in our results was not confusion between depression and anxiety, but insufficient adjustment when symptoms became more persistent and functionally impairing. This suggests that current systems may be better at recognizing the presence of distress than at recalibrating contextual interpretation, psychosomatic balance, and help-seeking guidance as clinical severity increases. Such a pattern is unsurprising. Large language models are highly capable of generating supportive text, but proportional escalation requires something closer to clinical judgment: recognizing that the same coping advice that is reasonable for mild distress may be inadequate when functioning is deteriorating.

This severity-calibration problem deserves more attention than it currently receives. The most obvious safety evaluations focus on crisis situations, especially explicit suicidality (16–19). Yet many clinically important failures occur earlier, in the transition from understandable distress to symptoms that warrant professional assessment. A model need not give dangerous advice to be clinically inadequate; it may simply remain too reassuring, too generic, or too self-management oriented as the presentation becomes more serious. Such subtle under-escalation may constitute an important failure mode even in the absence of dramatic safety errors and therefore warrants specific attention in future real-world evaluation.

This has implications for how AI should be positioned within digital mental health. Our findings suggest a potential role for general-purpose chatbots as low-threshold supportive tools, particularly for recognizing distress and providing initial psychologically supportive guidance. However, the present benchmarking study did not assess patient outcomes, help-seeking behavior, therapeutic alliance, or clinical effectiveness. Any adjunctive role should therefore be regarded as a potential application requiring prospective clinical evaluation rather than an established benefit. Such systems should remain complementary to, rather than substitutes for, psychotherapy, diagnosis, or clinical triage. The critical boundary is whether they can appropriately recognize when supportive guidance is no longer sufficient and professional assessment should be recommended. Recent reviews similarly situate conversational AI within broader supportive-care and self-management pathways, including diabetes self-management and nursing support in palliative care, while emphasizing the importance of reliability, clinical validation, and appropriate integration with professional care (56, 57).

For developers, the results suggest that future evaluation should move beyond broad mental-health benchmarks toward contextual stress tests. Models should be challenged with clinically equivalent psychological symptoms embedded in different chronic illnesses, with parallel scenarios varying wording while preserving construct and severity. Evaluation should also test whether recommendations scale appropriately with worsening functional impairment and whether the model can manage uncertainty when somatic and psychological explanations overlap. These are more demanding criteria than factual correctness, but they are closer to the problems that arise in actual chronic-illness care.

For clinicians, the relevance is different. General-purpose AI is likely to become part of the informal interpretive environment in which patients make sense of illness. Patients may use these systems not only to obtain information, but to decide whether their distress is normal, whether it reflects their disease, and whether they should seek help. The clinical question is therefore unlikely to be whether patients should use AI at all, but how its use can be incorporated into conversations about symptom interpretation, uncertainty, and help-seeking without granting the system more authority than its performance warrants.

The study also has implications for research design. If AI systems are increasingly proficient at producing superficially supportive responses, then future work must distinguish between linguistic plausibility and clinical adequacy. The latter requires evaluation of context, proportionality, and reproducibility. Patient-level studies will ultimately be needed to determine whether AI-mediated support improves distress, accelerates help-seeking, or changes engagement with care (30, 31, 48). Benchmarking cannot establish those outcomes, but it can identify where prospective evaluation is most necessary and where current systems remain most likely to fail.

Several features of the present design strengthen these inferences. The use of matched cross-condition scenarios allowed illness context to vary while holding psychological presentation broadly constant, and parallel scenario variants reduced dependence on any single prompt formulation. Blinded rating and repeated testing further separated average model performance from response stability. At the same time, the study remains a controlled simulation. Real patients disclose information incompletely, change topics, respond to reassurance, and engage in multi-turn exchanges that may alter both the quality and risk of AI-generated support. The density of illness-specific cues was also not identical across psychological presentations, with anxiety scenarios containing more explicit disease-related concerns than depression scenarios. Although this asymmetry was mirrored across T2DM and CSU and the cross-condition analyses paired scenarios within the same presentation, severity level, and parallel variant, it may have influenced the absolute ease with which models demonstrated chronic-illness contextualization and should therefore be considered when interpreting domain-specific findings. Only two chronic illnesses and three AI systems were examined, and commercial models evolve rapidly. Expert-rated appropriateness also cannot substitute for patient-perceived usefulness, therapeutic alliance, or clinical outcomes.

The broader conclusion is therefore not that general-purpose AI succeeds or fails at psychological support in chronic illness. Rather, the findings suggest that the frontier has moved. Basic emotional recognition is no longer the most informative test of model capability. The harder problem is whether AI can formulate distress in context, preserve appropriate uncertainty between psychological and somatic explanations, and adjust its recommendations as clinical severity changes. Those capacities-not conversational fluency alone-will determine whether general-purpose AI can become a meaningful component of digital mental-health support for people living with chronic disease.

## 5 Conclusion

The clinical promise of general-purpose AI in chronic-illness mental health support will not be determined by its ability to recognize distress or generate empathic language alone. The more consequential test is whether it can interpret psychological symptoms in relation to the illness that gives them meaning, preserve appropriate uncertainty when somatic and psychological explanations overlap, and alter its guidance as clinical need changes. Our findings suggest that current systems can often provide plausible first-contact psychological support, but remain less dependable when support requires contextual formulation, management of psychosomatic uncertainty, or severity-sensitive escalation. This distinction defines an important boundary between conversational competence and clinically meaningful support. As AI becomes increasingly embedded in how patients understand and respond to chronic illness, evaluation should therefore shift from asking whether models can produce plausible mental-health responses to asking whether those responses are appropriately contextualized, proportionate, and reproducible. Crossing that boundary will be essential before general-purpose AI can be regarded as more than an accessible conversational interface within digital mental health.

## Data Availability

The original contributions presented in the study are included in the article/**Supplementary Material**. Further inquiries can be directed to the corresponding authors.
